# Supplementary material for: Identification of druggable targets from the interactome of the Androgen Receptor and Serum Response Factor pathways in prostate cancer
Source: PLoS One. 2024 Dec 13;19(12):e0309491. doi: 10.1371/journal.pone.0309491 (PMC11642960; doi:10.1371/journal.pone.0309491)
Supplement: S1 Table — Numbers indicate LFQi values. Abbreviations: AR KD, AR knocked down; AR ENDO, endogenous AR. (DOCX) [file pone.0309491.s001.docx]

**Supplemental table 1. List of peptides precipitated with AR. Numbers indicate LFQi values.**

Abbreviations: AR KD, AR knocked down; AR ENDO, endogenous AR;
