# Supplementary material for: Identification of druggable targets from the interactome of the Androgen Receptor and Serum Response Factor pathways in prostate cancer
Source: PLoS One. 2024 Dec 13;19(12):e0309491. doi: 10.1371/journal.pone.0309491 (PMC11642960; doi:10.1371/journal.pone.0309491)

Figure 2 A

Blotted with AR (100kDa)

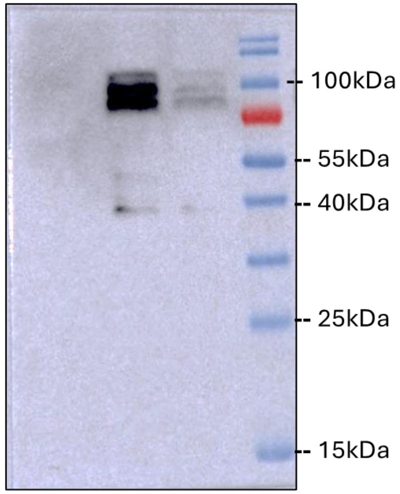

Blotted with GAPDH (36kDa)

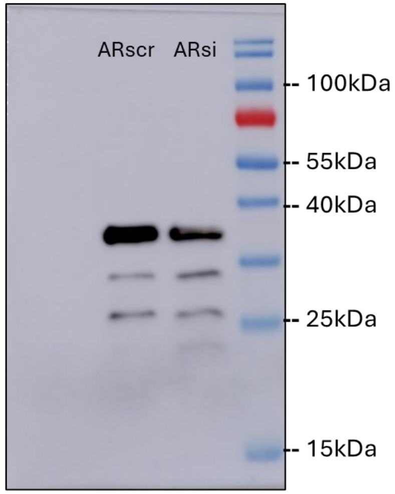

Figure 2 C

Blotted with SRF (67kDa)

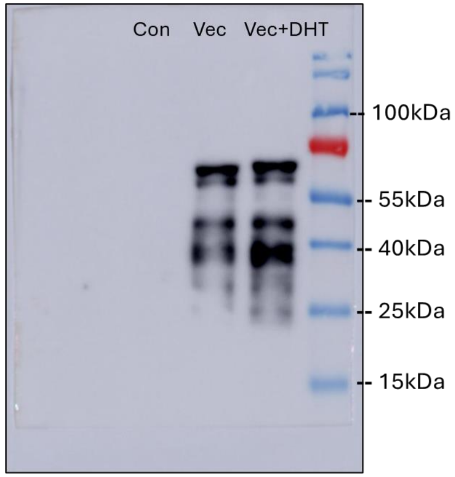

Blotted with GAPDH (36kDa)

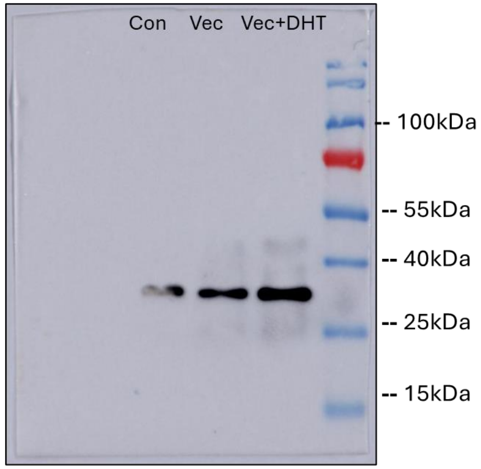

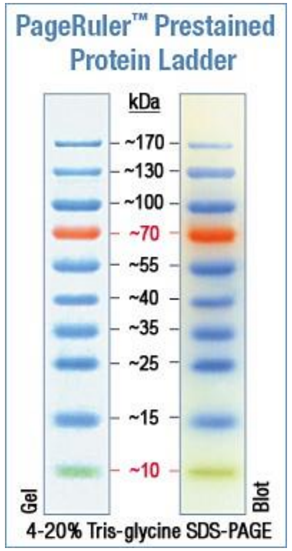

Figure 2 B

Blotted with SRF (67kDa

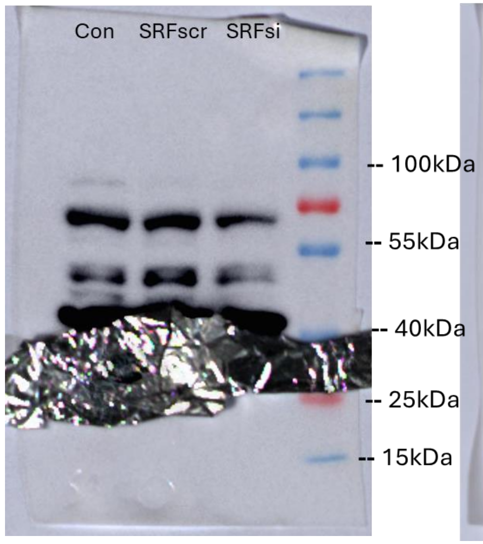

Blotted with GAPDH (36kDa)

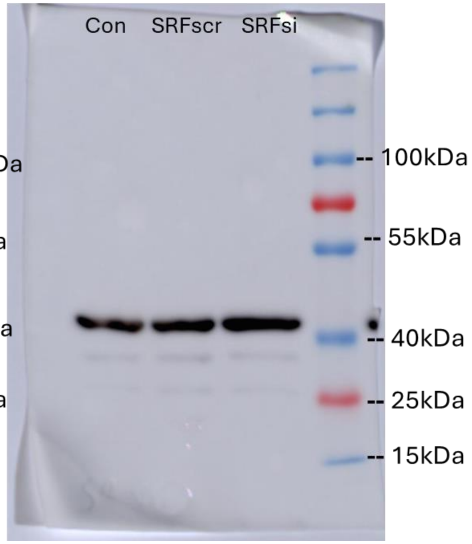

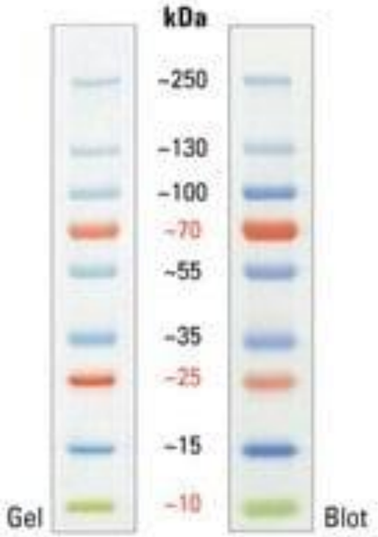

Supplement: S1 Raw images — (PDF) [file pone.0309491.s003.pdf]
